# Impact of Rheumatic Process in Left and Right Ventricular Function in Patients with Mitral Regurgitation

**DOI:** 10.5334/gh.1192

**Published:** 2023-03-17

**Authors:** Estu Rudiktyo, Emir Yonas, Maarten J. Cramer, Bambang B. Siswanto, Pieter A. Doevendans, Amiliana M. Soesanto

**Affiliations:** 1 Department of Cardiology and Vascular Medicine, Faculty of Medicine, Universitas Indonesia–National Cardiovascular Center Harapan Kita, Jakarta, Indonesia; 2 Department of Cardiology, Division Heart and Lungs, University Medical Center Utrecht, Utrecht, the Netherlands; 3 Central Military Hospital Utrecht, the Netherlands; 4 Netherlands Heart Institute Utrecht the Netherlands

**Keywords:** rheumatic heart disease, mitral regurgitation, ventricular dysfunction, impact

## Abstract

**Background:**

Mitral regurgitation (MR) burdens the left and right ventricles with a volume or pressure overload that leads to a series of compensatory adaptations that eventually lead to ventricular dysfunction, and it is well known that in rheumatic heart disease (RHD) that the inflammatory process not only occurs in the valve but also involves the myocardial and pericardial layers. However, whether the inflammatory process in rheumatic MR is associated with ventricular function besides hemodynamic changes is not yet established.

**Purpose:**

Evaluate whether rheumatic etiology is associated with ventricular dysfunction in patients with chronic MR.

**Methods:**

The study population comprised patients aged 18 years or older included in the registry who had echocardiography performed at the National Cardiovascular Center Harapan Kita in Indonesia during the study period with isolated primary MR due to rheumatic etiology and degenerative process with at least moderate regurgitation.

**Results:**

The current study included 1,130 patients with significant isolated degenerative MR and 276 patients with rheumatic MR. Patients with rheumatic MR were younger and had a higher prevalence of atrial fibrillation and pulmonary hypertension, worse left ventricle (LV) ejection fraction and tricuspid annular plane systolic excursion (TAPSE) value, and larger left atrium (LA) dimension compared to patients with degenerative mitral regurgitation (MR). Gender, age, LV end-systolic diameter, rheumatic etiology, and TAPSE were independently associated with more impaired LV ejection fraction. Whereas low LV ejection fraction, LV end-systolic diameter, and tricuspid peak velocity (TR) peak velocity >3.4 m/s were independently associated with more reduced right ventricle (RV) systolic function (Table 3).

**Conclusions:**

Rheumatic etiology was independently associated with more impaired left ventricular function; however, rheumatic etiology was not associated with reduced right ventricular systolic function in a patient with significant chronic MR.

## Introduction

Rheumatic heart disease (RHD) and its complications are significant health care problems in middle- and low-income countries because of very high prevalence and its tendency to affect patients at a productive age. According to the data published by the Global Burden of Disease (GBD) in 2015, at least 33.4 million people worldwide suffer from RHD [1]. This is significantly higher than the global prevalence of tuberculosis in 2018, accounting for approximately 10 million people [2]. Mitral regurgitation (MR) is one of the most common lesions in rheumatic heart disease besides mitral stenosis [3,4,5].

MR burdens the left ventricle with a volume load that leads to a series of left ventricular (LV) compensatory adaptations and adjustments that vary considerably during the prolonged clinical course of MR. The early compensatory changes with the utilization of the Frank-Starling mechanism are gradually replaced by a chronic remodeling process with enlargement of the LV chamber [6,7]. Eventually, these compensatory adaptations fail, LV dysfunction develops, and transition to a decompensated phase of chronic MR occurs [8]. At a later stage, right ventricle (RV) function may also decrease due to pulmonary hypertension. Both LV and RV dysfunction are independent predictors of poor outcomes in these patients [9].

In acute rheumatic fever (ARF), which is a precursor to RHD, the inflammatory process not only occurs in the valve but also involves the myocardial and even pericardial layers. Hence, the inflammatory process in ARF is also often referred to as pancarditis [10]. Myocardial involvement is confirmed by the finding of Aschoff nodules in the myocardium [11]. These nodules are also found in cardiac valve tissue. Aschoff nodules are pathognomonic findings in RHD. Another study using magnetic resonance imaging (MRI) showed myocardial fibrosis in patients with rheumatic mitral stenosis (MS) who did not have ischemic heart disease [12]. This finding shows that there was an inflammatory process in the myocardium that eventually led to fibrosis. This chronic inflammatory process might have a negative impact on ventricular function.

However, the association of this inflammatory rheumatic process and ventricular dysfunction in chronic MR have not been reported. Establishing this association is very important because this process was persistent even after mitral valve surgery. Therefore, the purpose of this study is to evaluate whether rheumatic etiology is associated with LV and RV systolic dysfunction in a patient with chronic MR.

## Methods

### Study design, subject and data collection

This study is a part of valve registry at the National Cardiovascular Center Harapan Kita (NCCHK). In brief, our valve registry was a retrospective, single-center, hospital-based registry of patients aged 18 years or older with valvular heart disease between January 2016 and June 2019 (42 months). The echocardiography result serves as the starting point for a subject to be admitted to the registry. If the report shows the presence of moderate or severe valve lesions, the patient is admitted to the registry, and detailed demographic and clinical characteristics are retrieved from their medical records and the hospital information system. The purpose of the registry is to provide information and describe characteristics of valvular heart disease in our hospital that served as national referral for cardiovascular disease.

In this study, patients with moderate or severe rheumatic MR were selected from the valve registry. As a comparison group, subjects with moderate to severe degenerative MR were also selected from the registry. Patients with concomitant structural lesions such as moderate or severe MS, aortic regurgitation or stenosis and pulmonary regurgitation or stenosis, congenital heart disease, or other structural heart diseases were excluded from the study. These abnormalities may also cause ventricular dysfunction that may confound interpretation of the study. Patients with moderate to severe tricuspid regurgitation were not excluded in this study. Hence, we systematically included patients with “isolated” significant MR in both groups. The purpose of selecting patients who also had degenerative MR was to include rheumatic etiology as one of the independent variables.

### Study site

NCCHK is a national referral hospital for cardiovascular disease in Jakarta, the capital city of Indonesia, and an academic hospital for the Faculty of Medicine, University of Indonesia.

### Echocardiographic analysis

Echocardiography was performed by sonographers, fellows, and residents and was reviewed by echocardiography consultants, echocardiography fellows, cardiology residents, and sonographers. All reports were reviewed by echocardiography consultants. In this study, echocardiography parameters were directly taken from the reports. Cutoff values for cardiac chamber dimension and left and RV contractility is based on American Society of Echocardiography (ASE) and European Association of Cardiovascular Imaging (EACVI) Recommendations for Cardiac Chamber Quantification by Echocardiography [13], while diagnosis and grading severities of valve abnormality were performed according to 2012 and 2017 European Society of Cardiology (ESC) Guidelines for the management of valvular heart disease [14,15]. The 2015 ESC Guidelines for the diagnosis and treatment of pulmonary hypertension (PH) are used for deciding the peak tricuspid regurgitation velocity cutoff value [16].

RHD was diagnosed using World Heart Federation (WHF) criteria, while degenerative MR was diagnosed using the definition from ASE [17,18]. Left ventricular ejection fraction (LVEF) was calculated according to the Simpson’s biplane method. From the apical four- and two-chamber views, the LV end-systolic and end-diastolic volumes were measured and LVEF was derived. An LVEF value of less than 60% is considered abnormal. Right ventricular function is expressed with tricuspid annular plane systolic excursion (TAPSE). TAPSE is measured using M-mode echocardiography in the apical four-chamber view to generate an image that illustrates the systolic longitudinal displacement of the lateral tricuspid annulus toward the apex. A value less than 17 mm is considered abnormal [13].

Severity of MR was assessed using a multiparametric integrated approach as recommended (qualitative, semiquantitative, and quantitative parameters), including vena contracta width and, when feasible, effective regurgitant orifice area and regurgitant volume calculated according to the proximal isovelocity surface area (PISA) method [14]. Moderate or severe MR is considered significant. Peak tricuspid regurgitation (TR) velocity, or TR Vmax, was assessed from multiple views, searching for the best envelope and maximal velocity. Peak TR velocity was measured from the spectral profile of the TR jet in the right ventricular inflow projection of the parasternal long-axis view, the parasternal short-axis view, or the apical four-chamber view. A peak TR velocity value of more than 3.4 m/s is considered as high probability of PH [16].

## Statistical Analysis

Continuous variables are expressed as mean ± standard deviation and categorical variables as frequency (percentage). Normality was established using the Shapiro-Wilk test. Continuous variables were compared between the rheumatic and degenerative MR groups using the Student’s t-test or the Mann-Whitney U test, as appropriate, whereas categorical variables were compared using the x^2^ test or Fisher’s exact test, as appropriate.

There are two dependent categorical variables in this study: (1) LV systolic function that is represented by LVEF (LVEF is reduced if less than 60%), and (2) RV systolic function that is represented by TAPSE that will be analyzed separately (TAPSE is reduced if less than 17 mm). The association between clinical and echocardiographic parameters with these dependent variables was evaluated using the logistic regression analysis, including as independent variables clinical and echocardiographic parameters associated in the univariable analysis with a *P* value <0.25. A *P* value of <0.05 was considered statistically significant. When analyzing the LVEF as the dependent variable, TAPSE was included as an independent variable, whereas when analyzing the TAPSE as the dependent variable, LVEF was included as an independent variable. Statistical analysis was performed with SPSS version 24 (SPSS, Inc., Chicago, IL).

## Results

### Baseline characteristics

In total, 1,130 patients with significant isolated degenerative MR and 276 patients with rheumatic MR were included in the current study. Baseline demographic characteristics, clinical, and echocardiographic characteristics are presented in Table 1. The mean age was 53 ± 13 years and 37 ± 14 years in the degenerative and rheumatic groups, respectively. Most patients with rheumatic MR were female (76.8%). Patients with rheumatic MR were younger and had a higher prevalence of atrial fibrillation (AF) and PH, worse LV ejection fraction and TAPSE value, and larger left atrium (LA) dimension compared with patients with degenerative MR.

**Table 1:** Baseline demographic characteristics, clinical, and echocardiographic characteristics (N = 1406).


PARAMETERS	ISOLATED MITRAL REGURGITATION DEGENERATIVE (N = 1130)	ISOLATED MITRAL REGURGITATION RHD (N = 276)	P

Sex			

Male	744 (65.8%)	64 (23.2%)	P < 0.0001

Female	386 (34.2%)	212 (76.8%)	

Age	53 ± 13	37 ± 14	P < 0.0001

EKG			

SR	732 (64.8%)	104 (37.7%)	P < 0.0001

AF	307 (27.2%)	145 (52.5%)	

Other	14 (1.2%)	4 (1.4%)	

LVEF			

(mean ± SD)	65.74 ± 11.43	61.51 ± 11.62	P < 0.0001

<60%	245 (21.7%)	88 (31.9%)	P < 0.0001

EDD	56.63 ± 8.57	57.7 ± 10.0	P = 0.154

ESD	35.60 ± 8.09	38.6 ± 8.4	P < 0.0001

LAVi			

(mean ± SD)	87.36 ± 70.37	193 ± 307	P < 0.0001

TAPSE			

(mean ± SD)	22.4 ± 5.5	20.5 ± 5.9	P < 0.0001

<17 mm	160 (14.2%)	68 (24.6%)	P < 0.0001

TR Vmax			

>3.4 m/s	262 (23.2%)	97 (35.1%)	P < 0.0001

Presence of at least moderate tricuspid regurgitation	254 (22.47%)	144 (52.2%)	P < 0.0001


Data are presented as n (%), mean ± SD, or median [IQR], as appropriate. AF = atrial fibrillation, EDD = end-diastolic diameter, ESD = end-systolic diameter, LAVi = left atrium volume index, LVEF = left ventricle ejection fraction, SR = sinus rhythm, TAPSE = tricuspid annular plane systolic excursion, TR Vmax = tricuspid regurgitation maximum velocity.

### Factors associated with LV dysfunction

Table 2 summarizes the results of the univariate and multivariable analyses for LV dysfunction. Gender, age, LV end-systolic diameter (ESD), and rheumatic etiology were independently associated with more impaired LV ejection fraction in a patient with chronic MR multivariable analysis.

**Table 2:** Univariable and multivariable analysis parameters associated with reduced LV ejection fraction (<60%).


	UNIVARIABLE ANALYSIS	MULTIVARIABLE ANALYSIS			
	
	*P* VALUE	Or	*P* VALUE	95% CONFIDENCE INTERVAL	

				LOWER	UPPER

Gender					

Female	Reference	0.31	0.000	0.177	0.560

Male	0.201				

Atrial fibrillation					

No	Reference	1.15	0.883	0.176	7.556

Yes	0.134				

Etiology					

Degenerative	Reference	2.54	0.012	1.223	5.265

Rheumatic	0.101				

TR Vmax					

≤3.4 m/s	Reference	0.37	0.370	0.824	1.682

>3.4 m/s	0.157				

TAPSE					

>17 mm	Reference	0.97	0.937	0.415	2.25

≤17 mm	<0.000				

Age (years)	0.056	0.96	0.000	0.943	0.983

ESD (mm)	<0.0001	0.76	0.000	0.726	0.802

LAVi (ml/m^2^)	0.651	–	–	–	–


ESD = end-systolic diameter, LAVi = left atrium volume index, LVEF = left ventricle ejection fraction, TAPSE = tricuspid annular plane systolic excursion, TR Vmax = tricuspid regurgitation maximum velocity.

### Factors associated with RV dysfunction

Table 3 shows the results of the univariate and multivariable analyses for RV dysfunction. Low LVEF, LV ESD, and peak TR velocity > 3.4 m/s were independently associated with RV systolic function measured as TAPSE in a patient with chronic MR in multivariable analysis. However, rheumatic etiology was not independently associated with lower TAPSE value in this population.

**Table 3:** Univariable and multivariable analysis parameters associated with impaired TAPSE (<17 mm).


	UNIVARIABLE ANALYSIS	MULTIVARIABLE ANALYSIS			
	
	*P* VALUE	OR	*P* VALUE	95% CONFIDENCE INTERVAL	

				LOWER	UPPER

Gender					

Female	Reference	0.90	0.688	0.547	1.488

Male	0.105				

Atrial fibrillation					

No	Reference	1.88	0.441	0.378	9.347

Yes	0.000				

Etiology					

Degenerative	Reference	1.19	0.622	0.594	2.390

Rheumatic	0.000				

TR Vmax					

≤3.4 m/s	Reference	0.60	0.001	0.441	0.805

>3.4 m/s	0.000				

LVEF					

≥60%	Reference	3.81	0.002	1.622	8.960

<60%	<0.000				

Age (years)	0.587	–	–	–	–

ESD (mm)	0.05	1.031	0.090	0.995	1.067

LAVi (ml/m^2^)	0.000	0.998	0.056	0.996	1.000


ESD = end-systolic diameter, LAVi = left atrium volume index, LVEF = left ventricle ejection fraction, TAPSE = tricuspid annular plane systolic excursion, TR Vmax = tricuspid regurgitation maximum velocity.

## Discussion

This is the first study that provides information on the association of the rheumatic process to the left and right ventricular systolic function in the patient with significant chronic MR. Patients with degenerative MR were included in this study as a comparison group to describe the association between the rheumatic process and reduced ventricular function independent from hemodynamic factors. We found that rheumatic etiology was independently associated with more impaired LV systolic function but not with RV systolic function. These findings suggest that besides the chronic volume overload of the left ventricle, there is another mechanism that worsens the LV function in patients with rheumatic MR. The intrinsic myocardial process in RHD may play a role in the mechanism of this impaired LV contractility [19].

The involvement of the myocardium in RHD has been well established in previous studies. Myocardial involvement is confirmed by the finding of Aschoff nodules in the myocardium and the cardiac valve tissue [11]. Aschoff nodules are pathognomonic findings in RHD. Another study showed that preoperative myocardial fibrosis was identified in 91.5% of MS patients [12]. Myocardial fibrosis identified by late gadolinium enhancement (LGE) protocol was a nonischemic pattern, and most of them were described as patchy myocardial fibrosis that varied across every segment of LV [12]. Su et al. also reported that rheumatic heart disease can induce congestive heart failure due to modulation of the p38 MAPK (mitogen-activated protein kinase) signal and cardiomyocyte apoptosis [20].

Interestingly, the association between these mechanisms and the impaired LV systolic function is not yet established. Williams et al. questioning the significance of myocarditis in children with acute rheumatic carditis [21]. Their study showed that despite serologic / laboratory evidence of ongoing systemic inflammation, levels of cardiac troponin I are not elevated in the serum of children with acute rheumatic carditis. This finding suggests that although myocardial inflammation may be present in acute rheumatic carditis, the extent of myocardial inflammation is not sufficient to cause myocardial injury that is signified by elevation of serum troponin I. Therefore, the extent of myocardial inflammation will not be sufficient to significantly affect myocardial performance in the absence of a significant valve lesion. Furthermore, Gaasch and Folland describe that LV dysfunction in MS can be explained without implicating a rheumatic myocardial factor [22]. In contrast, Beaton et al. reported that the global longitudinal strain (GLS) was decreased in subjects with RHD in the absence of abnormal systolic function compared to a normal subject [23]. Soesanto et al. showed that LV GLS had a moderate correlation with the volume of myocardial fibrosis by MRI in rheumatic MS [24]. However, the subject of these studies was mostly consisting of MS patients; therefore, direct comparison to a nonrheumatic MS group, which is very rare, was not available.

In this study, the difference of RV systolic function is not statistically significant between rheumatic and degenerative MR. Also, in multivariate analysis, we found that rheumatic etiology was not associated with more impaired RV systolic function. The explanation of this finding is unclear due to the lack of studies addressing this issue. A study by Pande et al. showed evidence of apoptosis in the RV of patients with rheumatic MS that occurred early during rheumatic valve disease, even with low RV systolic pressure [21]. However, whether this apoptosis was significant enough to cause RV dysfunction was not addressed further. Another possible explanation of this finding is the fact that the rheumatic process most commonly involves left-sided valves (aortic and mitral) than the right-sided valves [3,4,5]. Hence, the RV myocardium may be less affected by the rheumatic inflammatory process than the LV myocardium.

## Clinical Perspective

**Figure 1:**
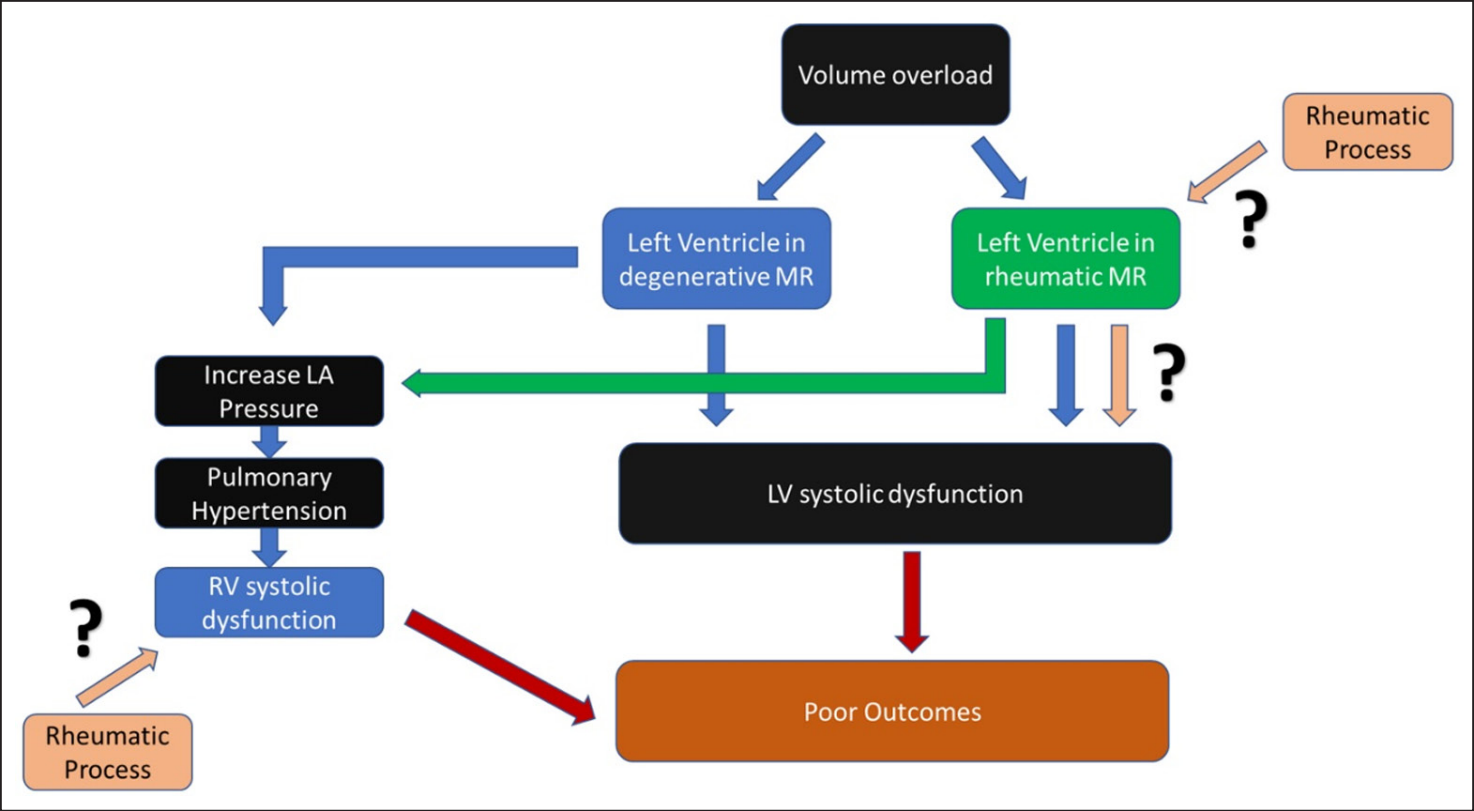
Central illustration. Proposed mechanism of left and right ventricular dysfunction in degenerative and rheumatic mitral regurgitation. Ventricular volume overload and pulmonary hypertension responsible for the development of left and right ventricular dysfunction, respectively, in chronic mitral regurgitation. However, the rheumatic process independently associated with the reduced ventricular function is not well established.

The current study gives new insight that there is an intrinsic process in patients with chronic rheumatic MR that significantly worsens LV systolic function in addition to volume overload (Figure 1). This process might continue after the corrective surgery for the MR, resulting in the persistence of decreased LV systolic function. Hence, the importance of secondary prophylaxis of rheumatic fever should be more emphasized in patients with chronic rheumatic MR. However, no specific study in this population is currently available to support this suggestion.

## Limitations

Although the present study is the first to identify the association between rheumatic etiology and the impaired LV and RV systolic function in patients with significant chronic MR, there are several limitations. First, the retrospective single-center nature of our study may constitute a major limitation. However, we believe that this should be considered a pilot study to enhance further research, including a larger subject, on this topic. Furthermore, we exclude coronary artery disease CAD as the possible mechanism of LV systolic dysfunction based only on clinical evidence and resting echo. Also, no data regarding the duration of MR was available. Duration of volume overload due to MR may impact the LV function. Finally, we only use LVEF and TAPSE as parameters for left and right ventricular function, respectively. However, both LVEF and TAPSE were established parameters of ventricular function and are widely used and have prognostic impact. Studies showed that myocardial deformation imaging of LV and RV were good parameters to identify subtle LV and RV dysfunction in RHD [25].

## Conclusion

Rheumatic etiology was independently associated with more impaired LV systolic function represented by LVEF; however it was not associated with reduced RV systolic function in patients with significant chronic MR.
